# Double NF1 Inactivation Affects Adrenocortical Function in NF1Prx1 Mice and a Human Patient

**DOI:** 10.1371/journal.pone.0119030

**Published:** 2015-03-16

**Authors:** Karolina Kobus, Daniela Hartl, Claus Eric Ott, Monika Osswald, Angela Huebner, Maja von der Hagen, Denise Emmerich, Jirko Kühnisch, Hans Morreau, Frederik J. Hes, Victor F. Mautner, Anja Harder, Sigrid Tinschert, Stefan Mundlos, Mateusz Kolanczyk

**Affiliations:** 1 Institute for Medical Genetics and Human Genetics, Charité, Universitätsmedizin Berlin, Berlin, Germany; 2 Max Planck Institute for Molecular Genetics, FG Development & Disease, Berlin, Germany; 3 Berlin-Brandenburg Center for Regenerative Therapies (BCRT), Berlin, Germany; 4 Department of Pathology, Leiden University Center, Albinusdreef 2, 2333ZA, Leiden, The Netherlands; 5 Department of Clinical Genetics, Leiden University Center, Albinusdreef 2, 2333ZA, Leiden, The Netherlands; 6 Department of Maxillofacial Surgery, University Hospital Eppendorf, Hamburg, Germany; 7 Institute of Neuropathology, University Hospital Münster, Münster, Germany; 8 Department of Medical Genetics, Molecular and Clinical Pharmacology, Medical University Innsbruck, Innsbruck, Austria; 9 Klinik für Kinder- und Jugendmedizin, Medizinische Fakultät Carl Gustav Carus, Technische Universität Dresden, Dresden, Germany; 10 Abteilung Neuropädiatrie, Medizinische Fakultät Carl Gustav Carus, Technische Universität Dresden, Dresden, Germany; Alexander Fleming Biomedical Sciences Research Center, GREECE

## Abstract

**Background:**

Neurofibromatosis type I (NF1, MIM#162200) is a relatively frequent genetic condition, which predisposes to tumor formation. Apart from tumors, individuals with NF1 often exhibit endocrine abnormalities such as precocious puberty (2,5–5% of NF1 patients) and some cases of hypertension (16% of NF1 patients). Several cases of adrenal cortex adenomas have been described in NF1 individuals supporting the notion that neurofibromin might play a role in adrenal cortex homeostasis. However, no experimental data were available to prove this hypothesis.

**Materials and Methods:**

We analysed Nf1Prx1 mice and one case of adrenal cortical hyperplasia in a NF1patient.

**Results:**

In Nf1Prx1 mice *Nf1* is inactivated in the developing limbs, head mesenchyme as well as in the adrenal gland cortex, but not the adrenal medulla or brain. We show that adrenal gland size is increased in NF1Prx1 mice. Nf1Prx1 female mice showed corticosterone and aldosterone overproduction. Molecular analysis of Nf1 deficient adrenals revealed deregulation of multiple proteins, including steroidogenic acute regulatory protein (StAR), a vital mitochondrial factor promoting transfer of cholesterol into steroid making mitochondria. This was associated with a marked upregulation of MAPK pathway and a female specific increase of cAMP concentration in murine adrenal lysates. Complementarily, we characterized a patient with neurofibromatosis type I with macronodular adrenal hyperplasia with ACTH-independent cortisol overproduction. Comparison of normal control tissue- and adrenal hyperplasia- derived genomic DNA revealed loss of heterozygosity (LOH) of the wild type *NF1* allele, showing that biallelic *NF1* gene inactivation occurred in the hyperplastic adrenal gland.

**Conclusions:**

Our data suggest that biallelic loss of *Nf1* induces autonomous adrenal hyper-activity. We conclude that *Nf1* is involved in the regulation of adrenal cortex function in mice and humans.

## Background

Neurofibromatosis type I (NF1) is a multi-system disease caused by loss-of-function mutations in the *NF1* gene which encodes a Ras-GAP protein, Neurofibromin. Primary disease manifestations are skin hyperpigmentations, so called ‘café au lait-spots’ (CALS) and freckles, benign cutaneous nerve sheath tumors (neurofibromas), and melanocytic iris hamartomas (Lisch nodules). NF1 is associated with skeletal manifestations including generalized osteopenia or osteoporosis, scoliosis and, less frequently, focal skeletal dysplasias (pseudarthrosis, sphenoid wing dysplasia). In addition, clinical manifestations include learning disabilitiesand an increased risk of malignancy [1–3]. Hypertension and precocious puberty (PP) are frequent complications in NF occurring in approximately 16% [4,5] and 2,5–5% [6–8] respectively. More than a half of hypertensive NF1 patients do not have one of the two well-known causes either renal artery stenosis or pheochromocytoma [9]. PP occurs mainly in association with optic pathway glioma (OPG) compromising hypothalamic and pituitary function; however, PP has been also reported in the absence of OPG [6–8]. Furthermore, PP is more commonly seen as a manifestation of NF1-associated OPG compared with isolated OPG; and an association between PP and NF1 itself has been concluded [6].

Neurofibromin ablation results in activation of canonical mitogen-activated protein kinase (MAPK) signalling. Dependent on the cellular context, this can also trigger activation of other pathways and downstream effectors including PI3K/mTOR [10], c-Jun/JNK [11] or JAK/STAT3 [12] signalling. Loss of neurofibromin was also shown to impact cAMP/PKA [13], Rho/ROCK/LIMK2/Cofilin [14] and Rac1/Pak1/LIMK1/Cofilin [15] pathways. *NF1* inactivation in the limb bud mesenchyme (Nf1Prx1) resulted in a phenotype with similarities to the NF1 tibial dysplasia, including reduced bone growth and long bone bowing [16]. Growth and bone formation defects were associated with deregulation of growth plate chondrocyte and osteoblast function, respectively [16]. Loss of Nf1 in mouse model in bone resulted in a profound defect of osteoblast differentiation causing defective matrix mineralization [16]. In addition to the osteoblast lineage, other mesenchymally-derived cell lineages are affected by loss of *Nf1*. In particular, we have shown that Nf1Prx1 mice have impaired skeletal muscle development [17]. This was associated with cell autonomous defect of myoblast differentiation and increased muscle connective tissue proliferation, resulting in muscle fibrosis [17]. Collectively these studies broadened our understanding of the mechanisms leading to musculoskeletal system manifestations in NF1 and revealed that they are caused by the primary changes in the affected cells rather than the effects of the nearby-localized tumors as previously postulated. However, some aspects of the NF1 pathology remain unexplained. For example, NF1 associates with hypertension and precocious puberty, aetiologies of which are not fully understood [18]. An endocrine involvement in the pathogenesis of these NF1 complications seems possible. Nf1 was previously implicated in the regulation of the pituitary-adrenal-endocrine axis [19]. Loss of *Nf1* in CNS progenitor cells (Nf1flox/flox x BLBPcre) resulted in a smaller pituitary gland and impaired hypothalamic growth hormone releasing hormone (GHRH) and growth hormone (GH) synthesis, while ACTH production was not affected [19]. Importantly, these defects could not be rescued by re-expression of the Ras regulatory GRD domain suggesting other than MAPK pathway (e.g. cAMP pathway) involvement. Inactivating mutations in *NF1* are frequent in adrenal medullary tumors (pheochromocytomas) and it has been shown that NF1 is required for control of proliferation and differentiation in chromaffin cells [20]. While medullary tumors are frequent in NF1, only several cases of adrenal cortex adenoma associated with NF1 have been described in the literature [21–23]. However, these cases of sporadic cortical hyperplasia suggest that NF1 might have yet unidentified roles in control of adrenocortical homeostasis. While *Nf1* is expressed in the embryonic adrenal cortex, its potential role in the adult adrenal cortex has not been investigated [24]. Here we present the analysis of Nf1Prx1 mice, as well as a NF1 patient with macronodular adrenal hyperplasia, indicating that Nf1 is indeed implicated in control of adrenal cortex function.

## Methods

### Animals

Nf1Prx1 mice were generated by breeding the Nf1flox/flox females with Nf1flox/wt/Prx1Cre males. All strains were maintained on the C57/Bl6 background. Nf1flox, Rosa26-LacZ and mT/mG mice were bred and genotyped as described previously [25–27]. Recombination efficiency in *Nf1* locus was tested in a competitive PCR using primers P1, P2 and P4 where P1 + P2 amplifies the excised allele (280 bp) and P1 + P4 amplifies the intact floxed allele (350 bp); for details see [17]. All animal experimental procedures were approved by the ‘Landesamt für Gesundheitsschutz und Technische Sicherheit (LaGeTSi), Berlin, Germany (protocol number ZH 120).

### qPCR

cDNAs were synthesized from 1 μg total RNAs with murine leukemia virus (MuLV) reverse transcriptase (Applied Biosystems, Carlsbad, CA). TaqMan universal PCR was then performed on an ABI PRISMs 7900 cycler (Applied Biosystems), using the SYBR green method according to the manufacturer’s instructions. Following primer sets were used:

Akr1b7: F 5′-CTGCCATTCTCAGCTTCAAC-3′; R 5′-CATGCACGGATCTCATCAAG-3′

Nf1ex41–42: F 5′-ggtggtgtgtatgtaaggtgttc-3′; R 5′-cccatgtggatttacacactaacc-3′

Cyp11B1: F 5′-GCAGAGGCAGAGATGATGC-3′; R 5′-ACAGGCCTGAAAGTGAGGAG-3′

StAR: F 5′-ACCTGCATGGTGCTTCATC-3′; R 5′-GGTTGGCGAACTCTATCTGG-3′

Cyp21A1: F 5′-GGATGAGATGGTTTGGGAAC-3′; R 5′-AGCACCACAAAGAGCTCCAG-3′

Hsd3b6: F 5′-AGACTGGGACTGCTGACACC-3′; R 5′-CTCCAGTTACCAGGCAGCTC-3′

Cyp11b2: F 5′-TTTCCAGCACCTAGCCTTTG-3′; R 5′-TAGGCCATCTGCACATCCTC-3′

Cyp11a1: F 5′-ATTGCGGAGCTGGAGATG-3′; R 5′-GCTTGAGAGGCTGGAAGTTG-3′

McR2: F 5′-GTGACAAAGCCAAGGAGAGG-3′; R 5′-GGTGTTTGCCGTTGACTTAC-3′

GAPDH: F 5′-AACTTTGGCATTGTGGAAGG-3′; R 5′-CAGTCTTCTGGGTGGCAGTG-3′

The expression levels were equilibrated towards GAPDH. Each determination was based on six biological and 18 technical replicates.

### Western blot

Whole adrenal gland lysates were prepared in RIPA buffer and protein concentration was determined with the BCA protein assay kit (Pierce). Ten micrograms of protein were loaded per lane and resolved by electrophoresis in SDS–polyacrylamide gels and transferred onto PVDF membranes (Amersham). For Western blot analysis, membranes were incubated with the following antibodies: anti-phospho-p42/44 (pERK1/2) #9102 (Cell Signaling), anti-p44 (ERK1) #4372 (Cell Signaling), anti-glycerinaldehyd-3-phosphat-dehydrogenase #Sc-25778 (Santa Cruz), anti-pStAR [28] (a generous gift from Dr. Steven R King, Baylor Collage of Medicine, Houston, TX, USA), anti-total StAR #sc-25806 (Santa Cruz). 2D gel electrophoresis was done as described previously [29]. Following secondary antibody incubation blots were developed with the Western Lightning Plus–ECL system (PerkinElmer).

### Histological anlysis

For histological analysis adult adrenal glands were fixed in 4% paraformaldehyd (PFA), dehydrated and embedded in paraffin. Eosin-Hamatoxilin and Heidenhain’s Azan trichrome staining was performed according to standard procedures.

### Immunohistological analysis

Paraffin sections were used for immunohistochemical analysis using Akr1b7 antibodies [30] provided by (Dr. Antoine Martinez—Clermont Université, France) and anti 20-alpha-HSD antibody from (Dr. Yacob Weinstein—Ben Gurion University, Israel).

### ELISA measurements

Following ELISA kits were used: Corticosterone ELISA kit ADI 900097 (Enzo Life Science); Aldosterone ELISA kit ADI 900173 (Enzo Life Science), ACTH ELISA kit 40–101–325001 (GenWay Biotech, Inc), cAMP EIA Kit Cat No 581001 (Cayman Chemical).

### Proteomics analyses

Adrenal glands were prepared from *Nf1*-deficient and control female mice (n = 6 per genotype). Proteins were extracted from shock-frozen tissue according to published protein extraction protocols [31]. Briefly, frozen sample buffer (50 mM TRIZMA Base, 50 mM KCl, and 20% w/v glycerol at pH 7.5 with freshly-added proteinase and phosphatase inhibitors (Complete&PhosStop, Roche Diagnostics) was added to the tissue samples. Samples were ground to fine powder in a mortar cooled by liquid nitrogen. After thawing samples to 4°C, they were sonicated on ice and incubated with DNAse. Protein extracts were supplied with 6.5 M urea, 2 M thiourea, 70 mM dithiothreitol, and 1% v/w of ampholyte mixture (Servalyte pH 2–4, Serva), and stored at-80°C until further use. Proteins were separated using the large-gel 2D-PAGE technique as described [31]. The gel format was 40 cm (isoelectric focusing) x 30 cm (SDS-PAGE) x 0.9 mm (gel width). Two dimensional protein patterns were obtained by silver staining of gels [31]. Protein spot patterns were evaluated by Delta2D imaging software (version 4.0, Decodon). Percent spot volumes (after background subtraction) were used for quantitative analysis of protein expression. The spot volume of each spot is the product of its size and intensity. Datasets were analyzed using a paired two-tailed Student’s t-test as *Nf1*-deficient and control samples were always handled in pairs starting with protein extraction until after completion of 2-D gel runs. Normality of data was elvaluated using Kolmogorov-Smirnov testing as described before elsewhere [31]. Only spot volume ratios ≥ 20% were considered (n = 6 biological replicates, significance threshold p ≤ 0.05) [31].

For protein identification by mass spectrometry (MS), 1.2 mg of the respective protein extracts were separated by 2D-PAGE and stained using a MS-compatible silver staining protocol [32]. Protein spots of interest were excised from 2D gels and subjected to in-gel tryptic digestion. Peptides were analyzed by an ESI-tandem-MS/MS on a LCQ Deca XP ion trap instrument (Thermo Scientific). Mass spectra were analyzed using MASCOT software (version 2.2) automatically searching the SwissProt database (513877 sequences). MS/MS ion search was performed with the following set of parameters: (i) taxonomy: Mus, (ii) proteolytic enzyme: trypsin, (iii) maximum of accepted missed cleavages: 1, (iv) mass value: monoisotopic, (v) peptide mass tolerance 0.8 Da, (vi) fragment mass tolerance: 0.8 Da, and (vii) variable modifications: oxidation of methionine and acrylamide adducts (propionamide) on cysteine. No fixed modifications were considered. Only proteins with scores corresponding to p<0.05 were considered. The cut-off score for individual peptides was equivalent to p<0.05 for each peptide as calculated by MASCOT.

### Analysis of human NF1 patient material.

The anonimised specimen of an NF1 patient with adrenal cortical hyperplasia was previously databased in the ebook www.hereditarypathology.org/. Patient’s tissue was handled with informed consent and according to the medical ethical guidelines described in the Code of Conduct for Proper Secondary Use of Human Tissue of the Dutch Federation of Biomedical Scientific Societies. No specific Institutional Review Board (IRB) approval was required as tissue samples were analyzed anonymously.

12 children with N1 were screened for adrenocortical dysfunction at a mean age of 8 years (min. 3 years, max. 14 years) during their routine diagnostic work up. None of the children had an opticus glioma within the region of the pituitary gland. The cortisol in blood in the morning before 9:00 am was screened and nine children had an ultrasound of the adrenal gland.

### DNA isolation from paraffin blocks

Adrenal gland paraffin blocks were sectioned at twenty μm and the region of interest (the cortical nodule) was dissected under inverted microscope. The collected tissue paraffin sections were deparafinised with xylene and rehydrated in the decreasing concentrations of ethanol. The tissue was lysed in 600μl of lysis buffer (proteinase K 0.3 mg/ml, 50 M Tris-HCl solution, 100mM NaCl, 100 mM EDTA, 1% SDS) over night at 52°C. Protein was precipitated with addition of 200 μl of 5M NaCl. The precipitates were centrifuged and the DNA solution collected in the new tubes. DNA was precipitated with addition of 350 μl of isopropanol and the pellet washed with 75% ethanol. DNA was dried and resolved in TE buffer.

### Sanger sequencing

Sanger sequencing was conducted as described previously [33]. Presence of LOH was interrogated using following primer pairs for amplification of the exon 1 of *NF1*;

NF1_1_MID8_fw: CTCCACAGACCCTCTCCTTG;

NF1_1_ MID8_rv: CTTCCCTTCCTTTCCTCCAG.

### LOH analysis by quantitative RT-PCR

To evaluate genomic DNA from adrenal tissue of NF1 patient for NF1-LOH, qPCR was performed as described in [34]. In brief, copy number for each amplicon was quantified relative to albumin (ALB) on 4q11 as autosomal two-copy control. In addition, we performed gender determination calculating the coagulation factor VIII (F8, Xq28) relative to our two-copy control, to assure its reliability. Primers used for LOH analysis:

RUNX2_6F AGACGGTCTCACTGCCTCTC

RUNX2_6R GATGGTCCCTAATGGTGTGG

F8-Ex3-F CTACCATCCAGGCTGAGGTTTATG

F8-Ex3-R CACCAACAGCATGAAGACTGACA

qh_CPD_1_F ATCCGCAGGAACAAGTGAGT

qh_CPD_1_R CATTGTCTGAGCGCCTACTG

qh_NF1-LOH-B_F TGAGCTGAAAAGGTATAGATCCTC

qh_NF1-LOH-B_R CCCCAAACACCGTAAGAGC

qh_NF1-LOH-J_F GTCGTGAAGGAAACCAGCAT

qh_NF1-LOH-J_R CCTGGTAGAAATGCGACTAAAGA

qh_NF1-LOH-Q_F CGCAACGGACCAGTTAAGA

qh_NF1-LOH-Q_R GCTCCTTTGTTCCTCCAACA

qh_RHOT1_1_F CTCCAGATGGAGAGGGTCAC

qh_RHOT1_1_R CCAGCGTAATGCTAGACACG

ALB-Ex12-F TGTTGCATGAGAAAACGCCA

ALB-Ex12-R GTCGCCTGTTCACCAAGGAT

## Results

### NF1Prx1 mice carry Nf1 inactivation in adrenal cortex but not adrenal medulla

**Fig 1:**
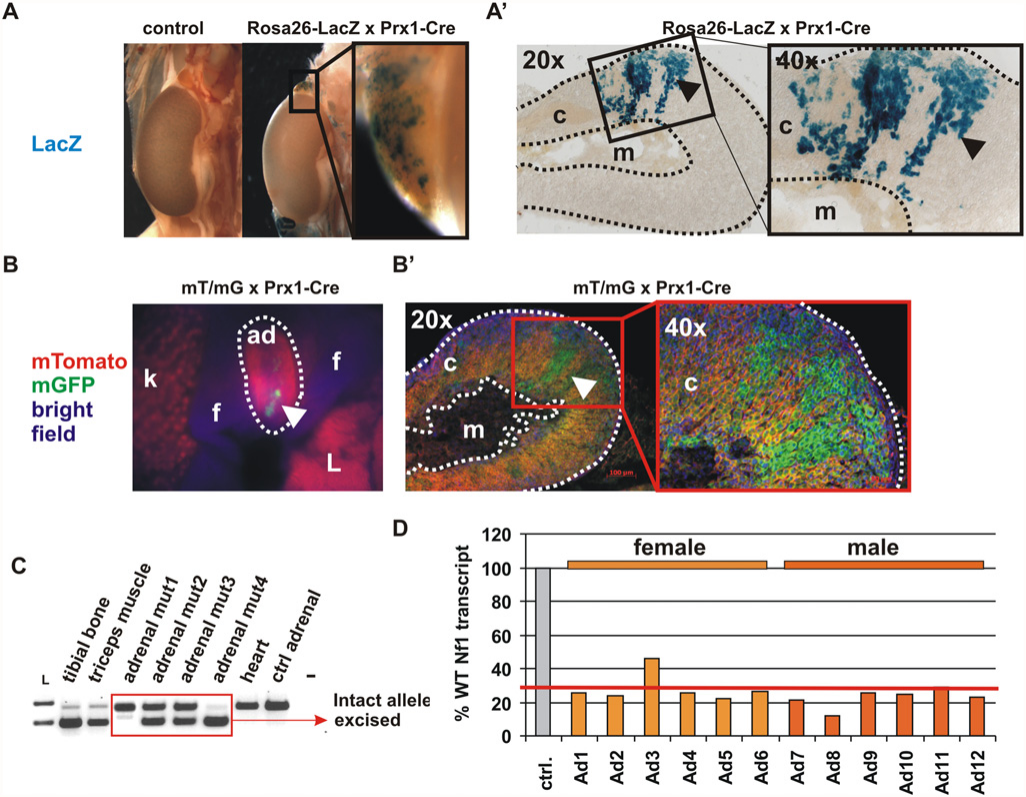
NF1 inactivation in the adrenal gland cortex. Whole mount LacZ staining showing a mosaic Cre-mediated recombination in adrenal cortex (dotted line) of 5-weeks-old Rosa26-lacZ reporter x Prx1-Cre mice. (B) Similar mosaic Cre recombination in the adrenal cortex visualized with mT/mG reporter mice. Note, no recombination was observed in the medulla. (C) PCR-based detection of recombined allele in the genomic DNA of 2-month-old Nf1Prx1 animals with varying degree of inactivation in the individual adrenal glands. Knock-out efficiency in the individual Nf1Prx1 adrenal glands assessed by quantitative RT-PCR. Note, out of 12 analysed mutant adrenal glands only one showed less than 30% efficiency of NF1 inactivation. c—cortex, m—medulla, f- fat, L- liver.

The Prx1-Cre transgene was previously reported to be expressed in the embryonic adrenal gland [35]. We tested expression of Cre recombinase in the adrenal gland by crossing Prx1-Cre mice with two reporter strains, Rosa26-LacZ [26] and mT/mG [27]. Both strains showed a patchy pattern of reporter activation in the adrenal cortex, but not in the medulla. The cortical expression was consistently present in all analysed reporter mice (Fig. 1A-B).

Importantly, reporters were not expressed in the hypothalamus or pituitary (data not shown). A varying degree of *Nf1* inactivation was observed in the individual adrenal glands on the genomic DNA level (Fig. 1C). We analysed NF1 transcript levels in individual adrenal glands from three mutant females (6 adrenal glands) and three mutant males (6 adrenal glands) as well as sex matched control mice (Fig. 1D). We did not detect significant differences in the knock-out efficiency between sexes. Out of 12 analyzed mutant adrenal glands only one showed less than 30% efficiency of NF1 inactivation (Fig. 1D). This data shows that in Nf1Prx1 mice Nf1 is inactivated in the adrenal gland cortex.

### Nf1Prx1 mice show adrenal hyperplasia ACTH independent hypercorticosteronemia and overproduction of aldosterone

**Fig 2:**
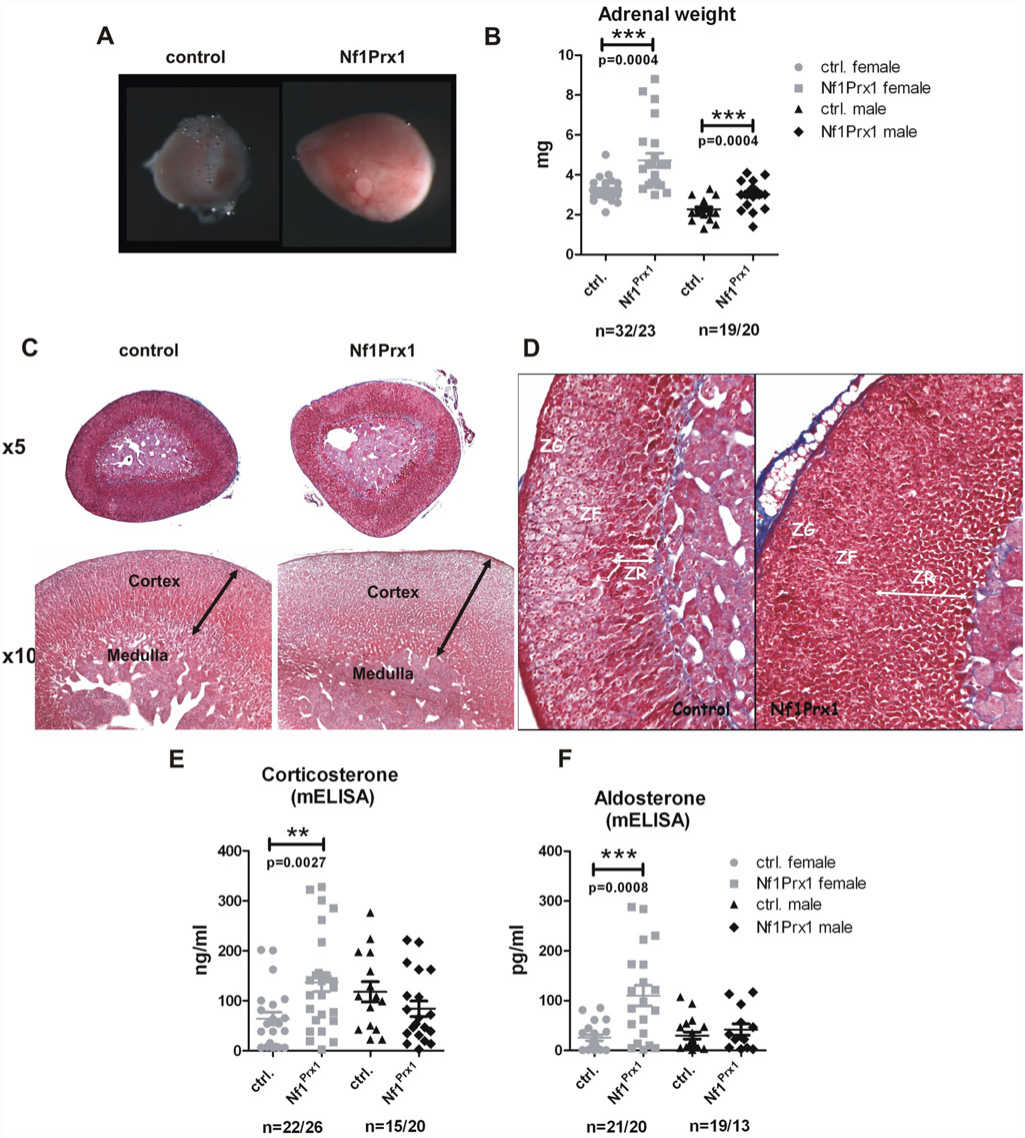
Increased weight of adrenal glands and defects of adrenal cortex zonation in the female Nf1Prx1 mice. (A) Macroscopic appearance of the representative female control and mutant adrenal glands. (B) Increased adrenal gland weight in female Nf1Prx1 mice. (C) Representative H&E staining of adrenal glands from 2-month-old female wt and Nf1Prx1 mice. Higher magnification illustrating, thicker and structurally irregular and disorganized adrenal cortex in comparison with control mice (lower panel). (D) Azan stained sections. Increased thickness of the (ZR) zona reticularis and (ZF) zona fasciculata in the adrenals of mutant Nf1Prx1 mice. (E, F, G) ELISA based quantitative analysis of the serum corticosterone and Aldosterone levels. Note a female specific increase of the corticosterone and Aldosterone level (measured 9–11 a.m.) Statistical evaluation was done with T-test with Welch’s correction.

We measured adrenal weight in two-month-old virgin mice. Adrenal size was significantly increased in mutant mice of both sexes as indicated by a weight increase (Fig. 2A, B).

Importantly, adrenal weight increase was more pronounced in female mutants than in male mutants (Fig. 2B). Histological analysis revealed inhomogeneous appearance of the zona fasciculata and zona reticularis with local increase of thickness of both these layers in mutant mice of both sexes (Fig. 2C, D). Both zona reticularis and zona fasciculata contained densely packed eosinophilic cells which were not aligned into cords. In contrast the zona glomerulosa appeared histologically unchanged (Fig. 2D).

We have previously shown using Azan staining muscle fibrosis in Nf1Prx1 model. Against expectation we did not observe fibrosis in adrenal glands of Nf1Prx1 mice. Consistent with the knock-out being restricted to the adrenal cortex, the histology of the medulla was not altered. These results revealed adrenal gland hyperplasia as a previously unrecognized phenotype in Nf1Prx1 mice. We next asked if adrenal gland hyperplasia in Nf1Prx1 mice also entails changes in steroid hormone synthesis. We measured plasma corticosterone levels using mouse corticosterone ELISA in samples from two-month-old mice of both sexes collected with care to minimize stress in a time window between 9 a.m. and 11 a.m. The corticosterone measurements revealed female specific hypercorticosteronemia in Nf1Prx1 mice (Fig. 2E). Importantly, ACTH levels measured in Nf1Prx1 females were unchanged with a tendency to decreased levels (88,82 ± 12,93 pg/mL in WT vs. 67,79 ± 5,281 pg/mL in Nf1Prx1), indicating that their basal hypercorticosteronaemia was independent of the pituitary and likely resulted from primary adrenal overactivity. The serum aldosterone levels were increased in female but not male Nf1Prx1 mice. Thus, similarly to glucocorticoids, mineralocorticoid synthesis was also affected by the loss of Nf1 (Fig. 2F). These findings corresponded with the more pronounced adrenal hyperplasia observed in female Nf1Prx1 mice compared to males.

### Nf1Prx1 mice show changes in the morphology of zona fasciculata and zona reticularis

**Fig 3:**
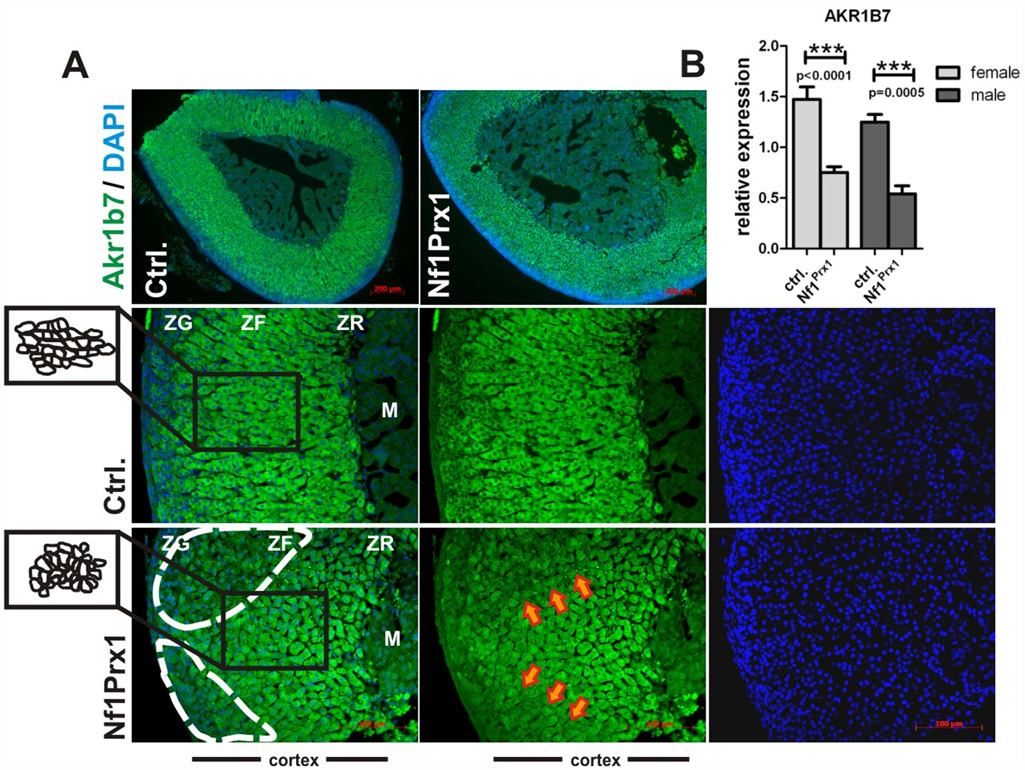
Changes in adrenal cortex morphology in Nf1Prx1 mice. (A) Expression of Akr1b7 labelling zona fasciculata and zona reticularis in the Nf1Prx1 adrenals. Paraffin sections of Nf1Prx1 and control adrenal glands immunostained for Akr1b7 (green) and counterstained with DAPI (blue). Note changes in the cell morphology of the zona fasciculata in mutant (graphically represented in the inset panels on the left) and abnormal expression pattern of Akr1b7 in the mutant cortex as compared to control specimens (white interrupted line and orange arrows). C.) qPCR measurement of the Akr1b7 gene expression in adrenal glands of Nf1Prx1. Expression was measured in six adrenal glands of female and male mice of both genotypes. T-test with Welch’s correction: 3 asterisks: p<0,001.

Nf1 has been implicated in the control of Ras, Rho/ROCK/LIMK2/Cofilin [14] and Rac1/Pak1/LIMK1/Cofilin signaling [15]. These pathways determine signalling events used by cells to sense changes in the extracellular environment. It is therefore interesting to note that cells in adrenal cortex of Nf1Prx1 mice (especially in the zona fasciculata) had altered morphology and failed to align into fascicles (Fig. 3A—see graphical depiction of cell shapes in the insert area).

This was visualized by immunostaining with anti-Akr1b7 antibody, which labelled both zona fasciculata and zona reticularis (Fig. 3A). We also noted that the expression of Akr1b7 protein in Nf1Prx1 adrenal cortex appeared unevenly distributed with markedly weaker immunolabeling in some areas of the zona fasciculata (Fig. 3A—interrupted line and orange arrows). This was confirmed by qPCR showing a decrease of Akr1b7 expression in male and female adrenal glands of Nf1Prx1 mice (Fig. 3B). A major function of Akr1b7 is to detoxify lipid peroxidation products created by oxidative deterioration of lipids containing carbon-carbon double bonds, especially those derived from polyunsaturated fatty acids (PUFAs) [36,37]. Therefore it appears likely that Nf1-deficient zona fasciculata cells have lower peroxylipid detoxification capacity. In contrast to changes in the morphology of zona fasciculata we did not detect overt changes in the thickness or morphology of the X-zone which was labelled with antibodies against 20-alpha-HSD (S1 Fig).

### Loss of Nf1 results in transcriptional deregulation of genes involved in corticosterone and aldosterone synthesis

**Fig 4:**
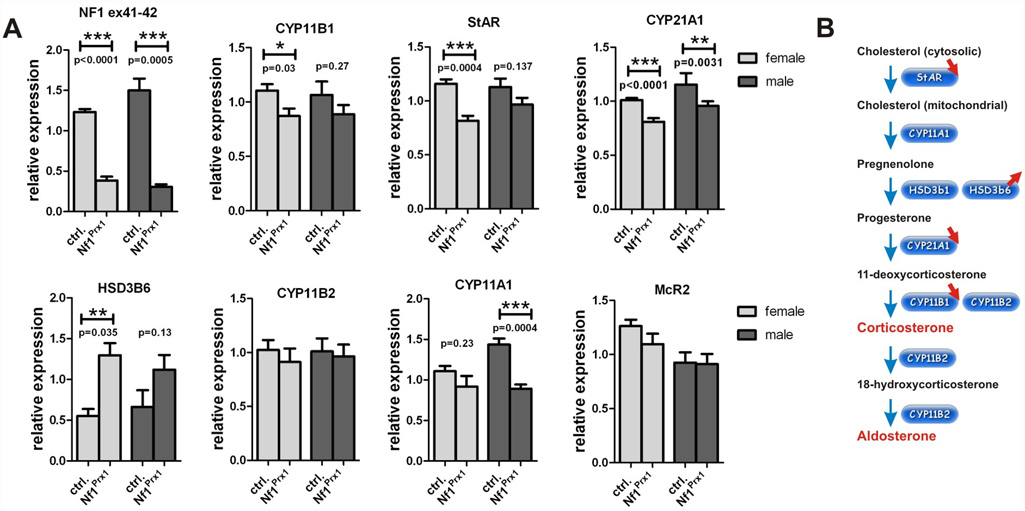
Changes in corticosteroid gene expression in adrenal glands of Nf1Prx1 mice. (A) qPCR based determination of the expression of genes involved in corticosteroid synthesis. Note decreased expression of StAR, Cyp11b1 and Cyp21a1 genes, which are involved in corticosterone synthesis and increased expression of Hsd3b6, the gene involved in aldosterone synthesis in female Nf1Prx1 adrenal glands. T-test with Welch’s correction: 1 asterisks: p<0.1; 2 asterisks: p<0.01; 3 asterisks: p<0.001. (B) A diagram of the corticosterone synthesis pathway depicting synthesis steps and major genes involved in the catalysis. Gene deregulations detected by the qPCR in the adrenal glands of Nf1Prx1 mice are indicated with red arrows.

Quantitative RT-PCR was used to analyze expression of genes involved in the corticosterone and aldosterone synthesis pathway in adrenal glands of Nf1Prx1 mice. In female mutant mice we detected a significant reduction of the transcript levels of Cyp11b1, Cyp21a1, and steroidogenic acute regulatory protein StAR, the genes involved in corticosterone synthesis (Fig. 4A).

Increased expression of HSD3b6 was seen, the gene encoding a protein crucially involved in aldosterone synthesis [38]. In male mutant mice we detected significantly decreased expression of Cyp11a1 and Cyp21a1, the genes involved in conversion of cholesterol and pregnenolone, respectively (Fig. 4B). Not regulated transcriptionaly were Cyp11b2, the gene encoding aldosterone synthase required for the final step of aldosterone synthesis [39]; HSD3b1 the zona fasciculata specific gene with function in both aldosterone and corticosterone synthesis (not shown); and McR2, the gene encoding ACTH receptor (Fig. 4A). Thus, at the transcriptional level several genes of the corticosterone synthesis pathway were down-regulated. The observed female-specific increase of HSD3b6 gene expression was in line with the increased synthesis of aldosterone in female mutants.

### Loss of *Nf1* causes increased MAPK signaling, increased StAR protein expression and increased StAR phosphorylation in the Nf1 deficient adrenal glands

**Fig 5:**
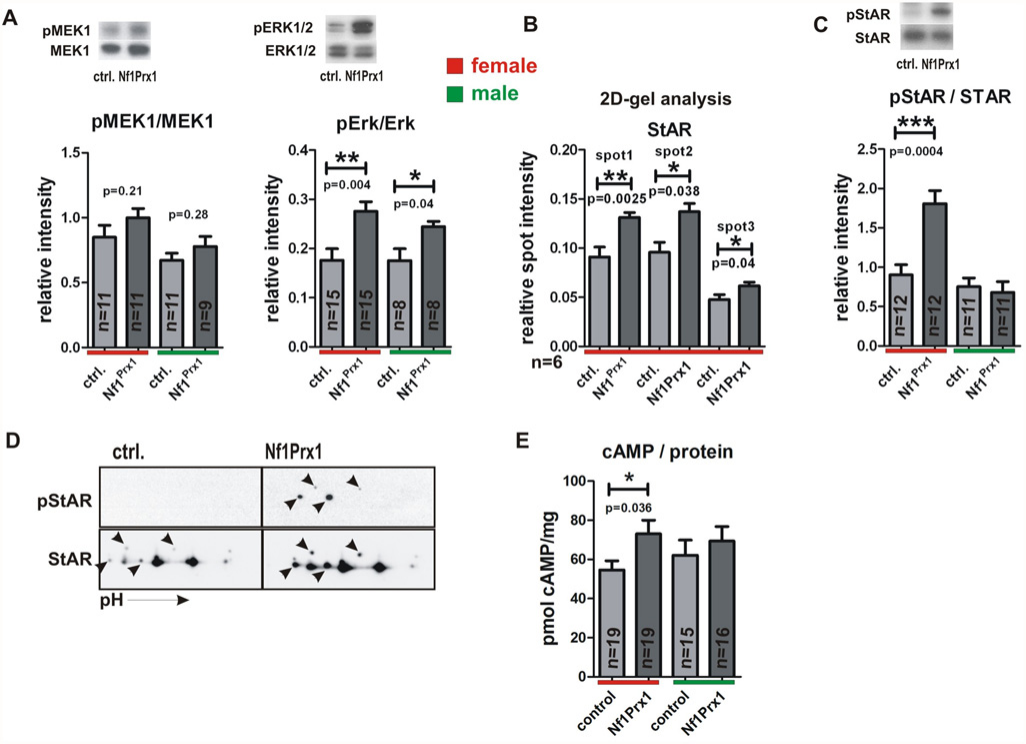
MAPK activation StAR overexpression and StAR hyperphosphorylation in the Nf1Prx1 female adrenal glands. A.) Densitometry based quantification of the western blots showing relative expression intensity of pMEK1 vs. MEK1 and pERK1/2 vs. ERK1/2. The upper inserts show representative bands of the female adrenal gland analysis. B.) Quantitative 2D-gel based analysis of the StAR expression level. 2D gels were performed with whole organ lysates of six control and six mutant murine adrenal glands. Relative spot intensity was determined with densitometry. The spots were identified with help of mass spectrometry as described in methods C.) Quantitative Western blot analysis of pStAR vs. StAR expression level in the adrenal glands from three months old Nf1Prx1 and control mice. The number of adrenal gland lysates used per genotype is given in the graph for each experiment. Band intensities were measured densitometrically and band intensity ratios were calculated. D.) 2D gel Western blot analysis of pStAR and StAR expression in female adrenal glands. Note increased phosphorylation of multiple StAR isoforms in the lysates of Nf1Prx1 adrenal glands. E.) ELISA determined concentrations of cAMP per mg of protein in the whole organ lysates from adrenal glands of three months old mice. T-test with Welch’s correction: 1 asterisks: p<0.1; 2 asterisks: p<0.01; 3 asterisks: p<0.001.

We measured MAPK pathway activation in the Nf1Prx1 adrenal glands using Western blots and densitometry. We detected a significantly increased level of ERK1/2 phosphorylation in the adrenal glands of NfPrx1 mutant mice of both sexes as compared to controls. In contrast MEK1 phosphorylation was not significantly increased (Fig. 5A).

To gain more information about proteome changes in the female Nf1Prx1 adrenal glands we conducted a 2-D gel based proteomic screen. Multiple proteins were found to be significantly deregulated in the mutant adrenals (S1 Table). Notably, we detected increased abundance of three protein spots identified as StAR (steroidogenic acute regulatory), a rate limiting enzyme of steroid synthesis (spot1/SID1586 ratio Mutant/Control = 1.44, p-value = 0.002; spot2/SID1574 ratio Mutant/Control = 1.43, p-value = 0.038; spot3/BID1176 ratio Mutant/Control = 1.29, p-value = 0.04) (Fig. 5A, see also S1 Table). Increased StAR protein expression was in contrast with transcriptional down-regulation of the StAR gene, which points to a possible deregulation of StAR protein turnover (Fig. 4A) [40]. In this context it is interesting to note that the expression of Lonp1, the mitochondrial protease responsible for StAR degradation [41] was found to be significantly decreased in mutant adrenals (spot SID343 ratio Mutant/Control = 0.63, p-value = 0.002; spot SID343 ratio Mutant/Control = 0.59, p-value = 0.02) (S1 Table).

To further explore the possible role of StAR in the observed phenotype we tested StAR phosphorylation status using pStAR specific antibodies [42]. StAR phosphorylation was previously shown to positively impact its catalytic activity [42]. Western blot analysis revealed a strong female-specific hyperphosphorylation of StAR in the Nf1Prx1 mice (Fig. 5C). Additionally, 2D-gel Western blot analysis revealed that multiple isoforms of StAR protein were hyperphosphorylated in the mutant mice (Fig. 5D). Thus, StAR phosphorylation was markedly increased in the adrenal glands of the Nf1Prx1 female mice, which is a likely cause of the observed increase of the corticoserone synthesis.

cAMP signaling has been implicated in the control of adrenocortical development and growth [43]. Measurement of the cAMP level in the whole adrenal gland lysates revealed a significantly increased cAMP concentration in the hyperplastic adrenal glands of mutant female mice (Fig. 5C).

### Adrenal macronodular hyperplasia with cortisol overproduction in NF1 patient associated with loss of heterozygosity in *NF1* locus

**Fig 6:**
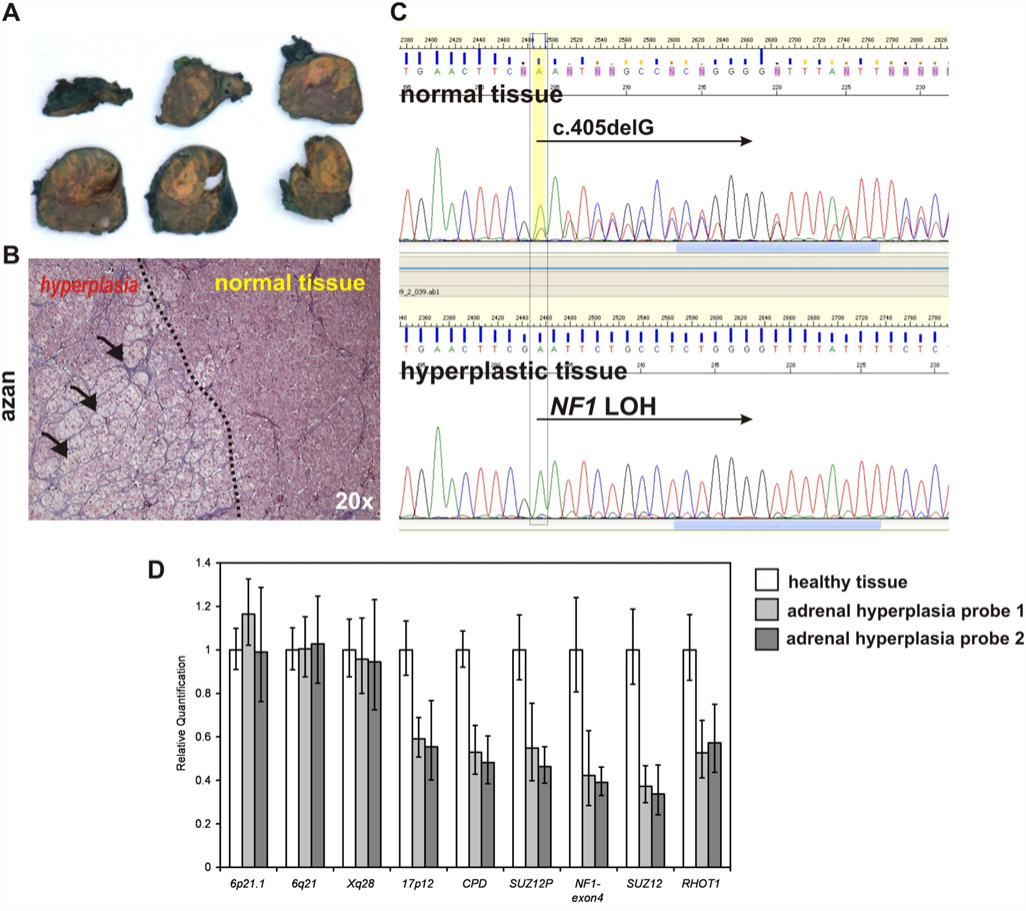
Loss of heterozygosity in NF1 locus associates with adrenal macronodular hyperplasia and cortisol overproduction in NF1 female patient. A.) Surgically removed adrenal gland of the 30 years old NF1 patient with yellow coloured adrenal hyperplasia. B.) Azan stained section of the adrenal tissue showing border between hyperplastic and healthy adrenal cortex tissue. C.) Sanger sequencing showing mixed sequences of the wt allele and c.405delG mutation in the blood (same in normally part of the adrenal gland) and a loss of heterozygosity detected in the hyperplastic part of the adrenal gland. D.) Real time quantitative PCR measuring gene copy number throughout chromosme 17 and on the control chromosomes. The mean values were calculated for each amplicon relative to albumin (ALB) on 4q11 as autosomal two-copy control. Error bars indicate the 95% confidence interval as given by the ABI 7900 SDS-Software. Results were calibrated to the mean values of a healthy control tissue (white bars). Decreased signal intensities were obtained in two hyperplastic adrenal tissue samples (grey bars) for all amplicons on chromosome 17 including amplicons for NF1 type1 deletions (CPD, RHOT1), NF1 type 2 deletions (SUZ12P, SUZ12), an amplicon localized upon the NF1 mutation detected by sequencing (NF1-exon4), and an amplicon on the short arm of chromosome 17 (17p12). In contrast, control amplicons localized on chromosome 6 (6p21.1, 6q21) and the X chromosome (Xq28) revealed normal signal intensities. These results indicate that a complete loss of chromosome 17 lead to NF1 loss of heterozygosity (LOH) detected by sequencing analysis. Copy-number analyses were repeated twice with similar results and confirmed using additional qPCR amplicons (not shown).

We analysed a female patient diagnosed with neurofibromatosis type 1 (NF1) and carrying the heterozygous *NF1* mutation NM_000267:c.405delG p.E8Nfs*16. The patient was diagnosed with a pilocytic astrocytoma of the cerebellum at the age of 20 and had multiple cutaneous neurofibromas. At the age of 30 years the patient presented with hypercortisolism, whereas metanephrines were not elevated. ACTH level was suppressed (ACTH < 5.0 ng/l) indicative of ACTH-independent Cushing syndrome. Upon surgical resection, the enlarged adrenal gland showed macronodular cortical hyperplasia with a dominant nodule of 1 cm in diameter. Tissue samples of the surgically removed adrenal gland were processed for paraffin embedding and DNA isolation. Adrenal hyperplastic tissue was isolated under inverted microscope from the paraffin sections (Fig. 6A, B).

DNA was isolated from paraffin sections as described in methods and Sanger sequencing revealed loss of heterozygosity in the hyperplastic adrenal tissue (see loss of wt allele in Fig. 6C). Quantitative PCR to determine the size of wt allele deletion revealed that the whole chromosome 17 was lost in the hyperplastic adrenal tissue, yielding biallelic *NF1* inactivation. Thus, somatic loss of both *NF1* alleles appears to underlie adrenal hyperplasia in the index patient. Additionally, we screened a small cohort of NF1 pediatric patients. In 12 children with NF1 morning cortisol levels and the volumetric measurement of the adrenal gland were within the normal range without signs of hyperplasia, suggesting that NF1 heterozygousity is not sufficient to induce the adrenocortical dysfunction.

## Discussion

This study unveils a new role of NF1 in the regulation of adrenocortical function. As shown by reporter mouse analysis, Prx1-cre transgene is active in the adrenal cortex but not adrenal medulla or pituitary gland. In Nf1Prx1 mice *Nf1* expression is impaired in adrenal gland leading to adrenal cortex hyperplasia with altered cellular morphology and deregulated gene and protein expression. The adrenal hyperplasia was associated with female specific overproduction of corticosterone and aldosterone while the ACTH level tended to decrease. Increased aldosterone synthesis was, at least in part, explained by the observed increased expression of type VI 3-beta-hydroxyl-steroid dehydrogenase gene (*Hsd3b6*) which is a functional counterpart of human *HSD3B1* gene [38]. Hsd3b6 is a crucial regulator of the aldosterone synthesis co-expressed in the zona glomerulosa with aldosterone synthase (*Cyp11b2*) [38]. Since *Hsd3b6* expression is under control of circadian clock and neurofibromin has been implicated in the control of circadian output in Drosophila [44], it is possible that expression of *Hsd3b6* is influenced by *Nf1* through MAPK regulated molecular clock mechanism. Indeed, we detected increased phosphorylation of ERK1/2 in the adrenals of female and male Nf1Prx1 mice indicating that Nf1 controls MAPK signaling intensity in adrenal cortex. ERK signaling has been previously shown to regulate corticosteroid synthesis and multiple endocrine and non-endocrine factors were shown to stimulate ERK1/2 phosphorylation in adrenocortical cells [45]. In line with this we found that Nf1Prx1 female mice had elevated corticosterone levels. Since pERK1/2 phosphorylation was increased in both females and males, but corticosterone synthesis was increased exclusively in females we reasoned that additional factors must contribute to the sex specificity of the glucocorticoid synthesis deregulation. At the transcriptional level most genes of corticosterone synthesis were down-regulated (Fig. 4). Our search for the pathogenic factor underlying hypercorticosteronemia led to the identification of StAR (steroidogenic acute regulatory protein) being overexpressed at protein level in the adrenal glands of female Nf1Prx1 mice (Fig. 5), an observation which was in contrast with *StAR* gene transcript down-regulation. This kind of discrepancy between mRNA and protein expression is not rare as abundance of many proteins is regulated at the level of protein stability [40]. Importantly, StAR controls the first and rate-limiting step of the glucocorticoid (GC) synthesis by shuttling cholesterol from outside mitochondria to the inner of the mitochondrial membranes [46]. Steroidogenic potential of StAR is regulated by phosphorylation of serine residues by protein kinase A (PKA) [42]. It has also been shown that StAR directly interacts and is phosphorylated by ERK, which stimulates GC synthesis [47,48]. Consistent with the observed female-specific corticosterone overproduction we detected increased levels of phospho-StAR in female but not male Nf1Prx1 adrenals (Fig. 5). The basis of this sex specificity remains elusive. One possible explanation is involvement of the female steroid-mediated signaling. The role of steroid hormones in the NF1 phenotype has been discussed previously in the context of tumorigenesis as number and size of neurofibromas in NF1 individuals has been shown to increase during pregnancy and puberty [49]. Several observations hint towards a possible role of steroid hormones in the context of adrenal function. First, female and male mice show significant differences in adrenal size such that at the age of eleven weeks the weight of adrenal glands in female mice is about two-fold higher compared to male littermates [50]. Second, in ovariectomised rodents, treatment with estrogen results in increased adrenal gland weight [51]. Lastly, estradiol treatment was reported to increase ERK1/2 phosphorylation in rat adrenal cortex cells and corticosteroid synthesis [52,53]. Thus, sex steroids impinge on adrenal growth and function and their potential role in the observed sex specificity of corticosterone and aldosterone overproduction in Nf1Prx1 mice should be addressed in future studies.

Apart from female specific StAR hyperphosphorylation, we detected female specific increase of cAMP concentration in the Nf1Prx1 adrenals. cAMP/PKA signaling has been previously implicated in the pathomechanism of NF1. In Schwann cells and osteoblasts, *NF1* inactivation leads to increased intracellular cAMP [54] and increased PKA activity respectively [55]. Excessive cAMP signalling in Schwann cells causes cell growth and impairs differentiation [56]. Similarly to Schwann cells, also adrenal cortex cells are sensitive to changes in cAMP signaling [43]. This is reflected by the genetics of corticotropin independent Cushing syndrome with adrenal hyperplasia (OMIM: #610489; #219080), which is observed in association with Carney complex (OMIM: #160980) as well as McCune-Albright syndrome (polyostotic fibrous dysplasia) (OMIM: #174800). Both conditions are caused by mutations in genes encoding proteins involved in cAMP signalling pathway. Carney complex is caused by inactivating germ line mutations in the *PRKAR1A* gene encoding regulatory type I-α (RIα) subunit of protein kinase A (PKA) [55]. McCune Albright syndrome is caused by somatic mutations in the *GNAS1* gene encoding the stimulatory G-protein alpha subunit, a key element of the signal transduction pathway linking receptor-ligand interactions with the activation of adenylate cyclase [57]. Sporadic cases of micronodular adrenal hyperplasia causing ACTH-independent Cushing syndrome were also linked to mutations in phosphodiesterase-11A (PDE11A) [58] and phosphodiesterase 8B (PDE8B) [59]. PDE11A is known to catalyze the hydrolysis of both cAMP and cyclic GMP (cGMP) whereas PDE8B is cAMP specific.

Thus, several lines of evidence indicate an important role of cAMP signalling in the aetiology of corticotrophin independent adrenal nodular hyperplasia. Moreover, the data presented in this manuscript support the importance of neurofibromin as a causative factor in adrenal nodular hyperplasia in patients with NF1. Biallelic inactivation of *NF1* had been previously reported in one case of adrenal cortical tumor [23], and adrenal adenoma have been described in association with NF1 in several reports [21–23].

## Conclusions

Our data suggest that previously observed adrenal adenoma cases in NF1 were not coincidental, and might have been caused by the biallelic inactivation of *NF1* in the adrenal cortex. It must be stressed that these cases are extremely rare. While there are no reports of a Cushing-like phenotype in children or adults with NF1, and the results of analysis in the small group of children with NF1 were normal, our data suggest that screening of NF1 population for potential subclinical adrenocortical hyperactivation should be undertaken, especially in the context of such symptoms as precocious puberty and hypertension.

## Supporting Information

S1 Fig Expression of 20-alpha-HSD labelling X zone. Paraffin sections of the adrenal glands from six months old Nf1Prx1 and control mice were immunostained for 20-alpha-HSD (green) and counterstained with DAPI (blue).
(TIF)

S1 Table Proteins significantly altered in adrenal glands of mutant and control female mice (n = 6 per genotype). (PDF)

## References

[1] Riccardi VM. Neurofibromatosis type 1 is a disorder of dysplasia: the importance of distinguishing features, consequences, and complications. Birth Defects Res A Clin Mol Teratol 2010; 88: 9–14. 10.1002/bdra.20616 19691086

[2] Gottfried ON, Viskochil DH, Couldwell WT. Neurofibromatosis Type 1 and tumorigenesis: molecular mechanisms and therapeutic implications. Neurosurg Focus 28: E8 10.3171/2010.3.FOCUS09324 20043723

[3] Korf BR Neurofibromatosis. Handb Clin Neurol 2013; 111: 333–340. 10.1016/B978-0-444-52891-9.00039-7 23622184

[4] Tedesco MA, Di Salvo G, Ratti G, Natale F, Calabrese E, Grassia C, et al Arterial distensibility and ambulatory blood pressure monitoring in young patients with neurofibromatosis type 1. Am J Hypertens 2001; 14: 559–566. 11411736 10.1016/s0895-7061(00)01303-0

[5] Lama G, Graziano L, Calabrese E, Grassia C, Rambaldi PF, Cioce F, et al Blood pressure and cardiovascular involvement in children with neurofibromatosis type1. Pediatr Nephrol 2004; 19: 413–418. 14991390 10.1007/s00467-003-1397-5

[6] Cnossen MH, Stam EN, Cooiman LC, Simonsz HJ, Stroink H, Oranje AP, et al Endocrinologic disorders and optic pathway gliomas in children with neurofibromatosis type 1. Pediatrics 1997; 100: 667–670. 9310522 10.1542/peds.100.4.667

[7] Virdis R, Sigorini M, Laiolo A, Lorenzetti E, Street ME, Villani AR, et al Neurofibromatosis type 1 and precocious puberty. J Pediatr Endocrinol Metab 2000; 13 Suppl 1: 841–844. 10969931 10.1515/jpem.2000.13.s1.841

[8] Habiby R, Silverman B, Listernick R, Charrow J. Neurofibromatosis type I and precocious puberty: beyond the chasm. J Pediatr 1997; 131: 786–787. 9403670 10.1016/s0022-3476(97)70117-3

[9] Ueda K, Awazu M, Konishi Y, Takenouchi T, Shimozato S, Kosaki K, et al Persistent hypertension despite successful dilation of a stenotic renal artery in a boy with neurofibromatosis type 1. Am J Med Genet A 2013; 161A: 1154–1157. 10.1002/ajmg.a.35829 23564656

[10] Johannessen CM, Reczek EE, James MF, Brems H, Legius E, Cichowski K. The NF1 tumor suppressor critically regulates TSC2 and mTOR. Proc Natl Acad Sci U S A 2005; 102: 8573–8578. 15937108 10.1073/pnas.0503224102 PMC1142482

[11] Daginakatte GC, Gianino SM, Zhao NW, Parsadanian AS, Gutmann DH. Increased c-Jun-NH2-kinase signaling in neurofibromatosis-1 heterozygous microglia drives microglia activation and promotes optic glioma proliferation. Cancer Res 2008; 68: 10358–10366. 10.1158/0008-5472.CAN-08-2506 19074905

[12] Banerjee S, Byrd JN, Gianino SM, Harpstrite SE, Rodriguez FJ, Tuskan RG, et al The neurofibromatosis type 1 tumor suppressor controls cell growth by regulating signal transducer and activator of transcription-3 activity in vitro and in vivo. Cancer Res 70: 1356–1366. 10.1158/0008-5472.CAN-09-2178 20124472 PMC5534849

[13] Dang I, De Vries GH. Aberrant cAMP metabolism in NF1 malignant peripheral nerve sheath tumor cells. Neurochem Res 36: 1697–1705. 10.1007/s11064-011-0433-2 21380540

[14] Ozawa T, Araki N, Yunoue S, Tokuo H, Feng L, Patrakitkomjorn S, et al The neurofibromatosis type 1 gene product neurofibromin enhances cell motility by regulating actin filament dynamics via the Rho-ROCK-LIMK2-cofilin pathway. J Biol Chem 2005; 280: 39524–39533. 16169856 10.1074/jbc.M503707200

[15] Starinsky-Elbaz S, Faigenbloom L, Friedman E, Stein R, Kloog Y. The pre-GAP-related domain of neurofibromin regulates cell migration through the LIM kinase/cofilin pathway. Mol Cell Neurosci 2009; 42: 278–287. 10.1016/j.mcn.2009.07.014 19666124

[16] Kolanczyk M, Kossler N, Kuhnisch J, Lavitas L, Stricker S, Wilkening U, et al Multiple roles for neurofibromin in skeletal development and growth. Hum Mol Genet 2007; 16: 874–886. 17317783 10.1093/hmg/ddm032

[17] Kossler N, Stricker S, Rodelsperger C, Robinson PN, Kim J, Dietrich C, et al Neurofibromin (Nf1) is required for skeletal muscle development. Hum Mol Genet 20: 2697–2709. 10.1093/hmg/ddr149 21478499 PMC3118757

[18] Zoller M, Rembeck B, Akesson HO, Angervall L. Life expectancy, mortality and prognostic factors in neurofibromatosis type 1. A twelve-year follow-up of an epidemiological study in Goteborg, Sweden. Acta Derm Venereol 1995; 75: 136–140. 7604643 10.2340/0001555575136140

[19] Hegedus B, Yeh TH, Lee da Y, Emnett RJ, Li J, Gutmann DH. Neurofibromin regulates somatic growth through the hypothalamic-pituitary axis. Hum Mol Genet 2008; 17: 2956–2966. 10.1093/hmg/ddn194 18614544 PMC2596853

[20] Burnichon N, Buffet A, Parfait B, Letouze E, Laurendeau I, Loriot C, et al Somatic NF1 inactivation is a frequent event in sporadic pheochromocytoma. Hum Mol Genet 2012; 21: 5397–5405. 10.1093/hmg/dds374 22962301

[21] Biagi P, Alessandri M, Campanella G, Castro R, Totteri A, Fanciullacci M. A case of neurofibromatosis type 1 with an aldosterone-producing adenoma of the adrenal. J Intern Med 1999; 246: 509–512. 10583721 10.1046/j.1365-2796.1999.00577.x

[22] Sartori P, Symons JC, Taylor NF, Grant DB. Adrenal cortical adenoma in a 13-year-old girl with neurofibromatosis. Case report and review of the literature. Acta Paediatr Scand 1989; 78: 476–478. 2500834 10.1111/j.1651-2227.1989.tb11116.x

[23] Gutmann DH, Cole JL, Stone WJ, Ponder BA, Collins FS. Loss of neurofibromin in adrenal gland tumors from patients with neurofibromatosis type I. Genes Chromosomes Cancer 1994; 10: 55–58. 7519874 10.1002/gcc.2870100109

[24] Daston MM, Ratner N. Neurofibromin, a predominantly neuronal GTPase activating protein in the adult, is ubiquitously expressed during development. Dev Dyn 1992; 195: 216–226. 1301085 10.1002/aja.1001950307

[25] Zhu Y, Romero MI, Ghosh P, Ye Z, Charnay P, Rushing EJ, et al Ablation of NF1 function in neurons induces abnormal development of cerebral cortex and reactive gliosis in the brain. Genes Dev 2001; 15: 859–876. 11297510 10.1101/gad.862101 PMC312666

[26] Soriano P. Generalized lacZ expression with the ROSA26 Cre reporter strain. Nat Genet 1999; 21: 70–71. 9916792 10.1038/5007

[27] Muzumdar MD, Tasic B, Miyamichi K, Li L, Luo L. A global double-fluorescent Cre reporter mouse. Genesis 2007; 45: 593–605. 17868096 10.1002/dvg.20335

[28] Manna PR, Chandrala SP, King SR, Jo Y, Counis R, Huhtaniemi IT, et al Molecular mechanisms of insulin-like growth factor-I mediated regulation of the steroidogenic acute regulatory protein in mouse leydig cells. Mol Endocrinol 2006; 20: 362–378. 16166197 10.1210/me.2004-0526

[29] Hartl D, Irmler M, Romer I, Mader MT, Mao L, Zabel C, et al Transcriptome and proteome analysis of early embryonic mouse brain development. Proteomics 2008; 8: 1257–1265. 10.1002/pmic.200700724 18283662

[30] Lefrancois-Martinez AM, Bertherat J, Val P, Tournaire C, Gallo-Payet N, Hyndman D, et al Decreased expression of cyclic adenosine monophosphate-regulated aldose reductase (AKR1B1) is associated with malignancy in human sporadic adrenocortical tumors. J Clin Endocrinol Metab 2004; 89: 3010–3019. 15181092 10.1210/jc.2003-031830

[31] Zabel C, Mao L, Woodman B, Rohe M, Wacker MA, Klare Y, et al A large number of protein expression changes occur early in life and precede phenotype onset in a mouse model for huntington disease. Mol Cell Proteomics 2009; 8: 720–734. 10.1074/mcp.M800277-MCP200 19043139 PMC2667354

[32] Nebrich G, Herrmann M, Sagi D, Klose J, Giavalisco P. High MS-compatibility of silver nitrate-stained protein spots from 2-DE gels using ZipPlates and AnchorChips for successful protein identification. Electrophoresis 2007; 28: 1607–1614. 17447244 10.1002/elps.200600656

[33] Baasanjav S, Jamsheer A, Kolanczyk M, Horn D, Latos T, Hoffmann K, et al Osteopoikilosis and multiple exostoses caused by novel mutations in LEMD3 and EXT1 genes respectively—coincidence within one family. BMC Med Genet 2010; 11: 110 10.1186/1471-2350-11-110 20618940 PMC2912259

[34] Ott CE, Leschik G, Trotier F, Brueton L, Brunner HG, Brussel W, et al Deletions of the RUNX2 gene are present in about 10% of individuals with cleidocranial dysplasia. Hum Mutat 2010; 31: E1587–1593. 10.1002/humu.21298 20648631

[35] Durland JL, Sferlazzo M, Logan M, Burke AC. Visualizing the lateral somitic frontier in the Prx1Cre transgenic mouse. J Anat 2008; 212: 590–602. 10.1111/j.1469-7580.2008.00879.x 18430087 PMC2409079

[36] Martinez A, Aigueperse C, Val P, Dussault M, Tournaire C, Berger M, et al Physiological functions and hormonal regulation of mouse vas deferens protein (AKR1B7) in steroidogenic tissues. Chem Biol Interact 2001; 130–132: 903–917. 11306105 10.1016/s0009-2797(00)00244-1

[37] Schneider C, Porter NA, Brash AR. Routes to 4-hydroxynonenal: fundamental issues in the mechanisms of lipid peroxidation. J Biol Chem 2008; 283: 15539–15543. 10.1074/jbc.R800001200 18285327 PMC2414272

[38] Doi M, Takahashi Y, Komatsu R, Yamazaki F, Yamada H, Haraguchi S, et al Salt-sensitive hypertension in circadian clock-deficient Cry-null mice involves dysregulated adrenal Hsd3b6. Nat Med 2010; 16: 67–74. 10.1038/nm.2061 20023637

[39] Domalik LJ, Chaplin DD, Kirkman MS, Wu RC, Liu WW, Howard TA, et al Different isozymes of mouse 11 beta-hydroxylase produce mineralocorticoids and glucocorticoids. Mol Endocrinol 1991; 5: 1853–1861. 1686470 10.1210/mend-5-12-1853

[40] Schwanhausser B, Busse D, Li N, Dittmar G, Schuchhardt J, Wolf J, et al Global quantification of mammalian gene expression control. Nature 2011; 473: 337–342. 10.1038/nature10098 21593866

[41] Granot Z, Kobiler O, Melamed-Book N, Eimerl S, Bahat A, Lu B, et al Turnover of mitochondrial steroidogenic acute regulatory (StAR) protein by Lon protease: the unexpected effect of proteasome inhibitors. Mol Endocrinol 2007; 21: 2164–2177. 17579211 10.1210/me.2005-0458

[42] Arakane F, King SR, Du Y, Kallen CB, Walsh LP, Watari H, et al Phosphorylation of steroidogenic acute regulatory protein (StAR) modulates its steroidogenic activity. J Biol Chem 1997; 272: 32656–32662. 9405483 10.1074/jbc.272.51.32656

[43] de Joussineau C, Sahut-Barnola I, Levy I, Saloustros E, Val P, Stratakis CA, et al The cAMP pathway and the control of adrenocortical development and growth. Mol Cell Endocrinol 2012; 351: 28–36. 10.1016/j.mce.2011.10.006 22019902 PMC3678347

[44] Williams JA, Su HS, Bernards A, Field J, Sehgal A. A circadian output in Drosophila mediated by neurofibromatosis-1 and Ras/MAPK. Science 2001; 293: 2251–2256. 11567138 10.1126/science.1063097

[45] Hoeflich A, Bielohuby M. Mechanisms of adrenal gland growth: signal integration by extracellular signal regulated kinases1/2. J Mol Endocrinol 2009; 42: 191–203. 10.1677/JME-08-0160 19052254

[46] Miller WL, Strauss JF 3rd. Molecular pathology and mechanism of action of the steroidogenic acute regulatory protein, StAR. J Steroid Biochem Mol Biol 1999; 69: 131–141. 10418987 10.1016/s0960-0760(98)00153-8

[47] Paz C, Poderoso C, Maloberti P, Cornejo Maciel F, Mendez C, Poderoso JJ, et al Detection of a mitochondrial kinase complex that mediates PKA-MEK-ERK-dependent phosphorylation of mitochondrial proteins involved in the regulation of steroid biosynthesis. Methods Enzymol 2009; 457: 169–192. 10.1016/S0076-6879(09)05010-1 19426868

[48] Poderoso C, Converso DP, Maloberti P, Duarte A, Neuman I, Galli S, et al A mitochondrial kinase complex is essential to mediate an ERK1/2-dependent phosphorylation of a key regulatory protein in steroid biosynthesis. PLoS One 2008; 3: e1443 10.1371/journal.pone.0001443 18197253 PMC2175533

[49] Roth TM, Petty EM, Barald KF. The role of steroid hormones in the NF1 phenotype: focus on pregnancy. Am J Med Genet A 2008; 146A: 1624–1633. 10.1002/ajmg.a.32301 18481270

[50] Bielohuby M, Herbach N, Wanke R, Maser-Gluth C, Beuschlein F, Wolf E, et al Growth analysis of the mouse adrenal gland from weaning to adulthood: time- and gender-dependent alterations of cell size and number in the cortical compartment. Am J Physiol Endocrinol Metab 2007; 293: E139–146. 17374700 10.1152/ajpendo.00705.2006

[51] Saruhan BG, Ozdemir N. Effect of ovariectomy and of estrogen treatment on the adrenal gland and body weight in rats. Saudi Med J 2005; 26: 1705–1709. 16311652

[52] Nowak KW, Neri G, Nussdorfer GG, Malendowicz LK. Effects of sex hormones on the steroidogenic activity of dispersed adrenocortical cells of the rat adrenal cortex. Life Sci 1995; 57: 833–837. 7630311 10.1016/0024-3205(95)02015-b

[53] Tron’ko MD, Kovzun OI, Mykosha OS. [The influence of protein kinases ERK, JNK and nuclear transcriptional factor c-JUN on corticotropin signal transduction in adrenocortical cells]. Ukr Biokhim Zh 2008; 80: 65–69. 18959029

[54] Kim HA, Ratner N, Roberts TM, Stiles CD. Schwann cell proliferative responses to cAMP and Nf1 are mediated by cyclin D1. J Neurosci 2001; 21: 1110–1116. 11160381 10.1523/JNEUROSCI.21-04-01110.2001 PMC6762237

[55] Kirschner LS, Carney JA, Pack SD, Taymans SE, Giatzakis C, Cho YS, et al Mutations of the gene encoding the protein kinase A type I-alpha regulatory subunit in patients with the Carney complex. Nat Genet 2000; 26: 89–92. 10973256 10.1038/79238

[56] Howe DG, McCarthy KD. Retroviral inhibition of cAMP-dependent protein kinase inhibits myelination but not Schwann cell mitosis stimulated by interaction with neurons. J Neurosci 2000; 20: 3513–3521. 10804191 10.1523/JNEUROSCI.20-10-03513.2000 PMC6772664

[57] Lumbroso S, Paris F, Sultan C. McCune-Albright syndrome: molecular genetics. J Pediatr Endocrinol Metab 2002; 15 Suppl 3: 875–882. 12199345

[58] Horvath A, Boikos S, Giatzakis C, Robinson-White A, Groussin L, Griffin KJ, et al A genome-wide scan identifies mutations in the gene encoding phosphodiesterase 11A4 (PDE11A) in individuals with adrenocortical hyperplasia. Nat Genet 2006; 38: 794–800. 16767104 10.1038/ng1809

[59] Horvath A, Mericq V, Stratakis CA. Mutation in PDE8B, a cyclic AMP-specific phosphodiesterase in adrenal hyperplasia. N Engl J Med 2008; 358: 750–752. 10.1056/NEJMc0706182 18272904

