# Supplementary figures and images for: Double NF1 Inactivation Affects Adrenocortical Function in NF1Prx1 Mice and a Human Patient
Source: PLoS One. 2015 Mar 16;10(3):e0119030. doi: 10.1371/journal.pone.0119030 (PMC4361563; doi:10.1371/journal.pone.0119030)

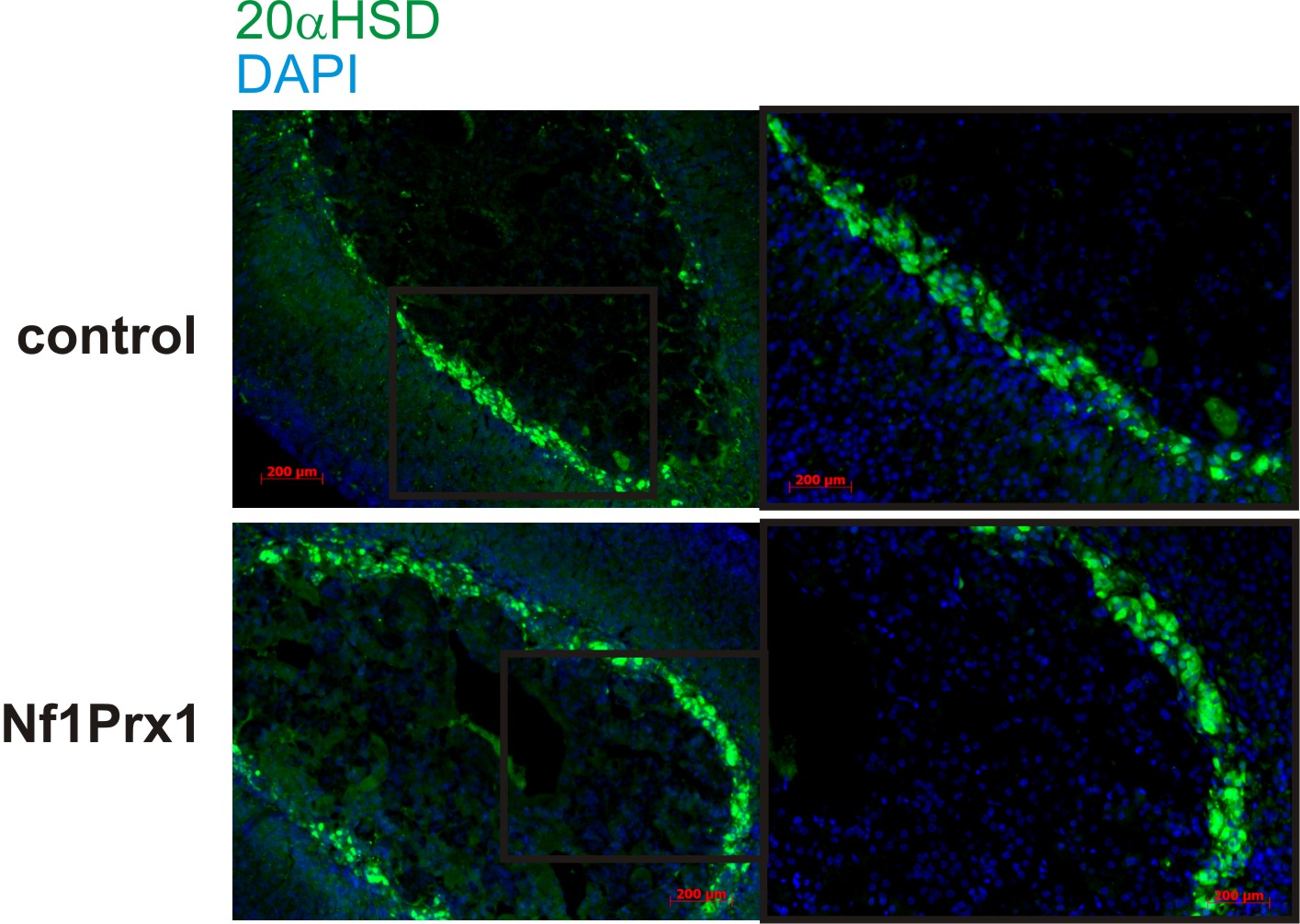

Supplement: S1 Fig — Paraffin sections of the adrenal glands from six months old Nf1Prx1 and control mice were immunostained for 20-alpha-HSD (green) and counterstained with DAPI (blue). (TIF) [file pone.0119030.s001.tif]
