# Supplementary material for: Double NF1 Inactivation Affects Adrenocortical Function in NF1Prx1 Mice and a Human Patient
Source: PLoS One. 2015 Mar 16;10(3):e0119030. doi: 10.1371/journal.pone.0119030 (PMC4361563; doi:10.1371/journal.pone.0119030)
Supplement: S1 Table — (PDF) [file pone.0119030.s002.pdf]

Spot ID	p-value	expression ratio (Mut/Ctrl)	MOWSE score	Number of identified peptides	Sequence coverage (%)	Gene name
BID770	0,02	0,64	188	3	8	Acads
BID763	0,01	1,68	54	1	2	Acat2
BID1747	0,04	1,60	66	1	8	Acot13
SID1527	0,04	1,59	149	3	10	Actb
SID1541	0,04	2,25	149	3	10	Actb
BID614	0,01	0,83	246	5	14	Ahcy
BID617	0,04	0,68	246	5	14	Ahcy
SID848	0,02	2,16	65	2	3	Alb
BID2482	0,04	1,46	39	1	1	Alb
BID563	0,01	1,90	107	2	4	Aldh9a1
BID451	0,00	1,67	111	2	3	Anxa11
BID353	0,00	0,56	104	2	4	Arcn1
BID991	0,02	0,58	409	8	25	Atp5c1
SID1146	0,01	1,40	126	3	16	Capza1
SID1444	0,03	1,67	283	5	29	Clic1
SID1246	0,02	1,67	157	4	15	Clta
BID1862	0,05	1,43	68	2	20	Cstb
BID477	0,00	0,64	37	1	2	Cyp11a1
BID526	0,00	0,57	67	2	6	Cyp11a1
BID504	0,00	0,70	165	2	6	Cyp11b2
BID436	0,03	1,44	131	3	10	D10Wsu52e
SID1240	0,05	1,48	280	5	20	Eef1d
BID611	0,04	0,67	144	3	8	Eif4a3
BID458	0,05	1,57	190	4	10	Fgb
SID1428	0,05	0,63	357	7	29	Glod4
SID1432	0,01	2,03	347	7	27	Glod4
SID1202	0,03	1,92	40	1	2	Gnb4
BID791	0,03	1,44	410	7	25	Hnrnpa3
BID772	0,00	1,63	102	2	6	Hnrnpd
SID661	0,00	0,66	104	1	4	Hspd1
SID666	0,03	0,52	55	1	4	Hspd1
SID952	0,03	2,74	44	1	1	Hspd1
SID476	0,04	0,61	77	2	2	Immt
SID1495	0,04	0,70	97	2	8	Impa1
BID689	0,02	0,70	264	6	13	Ivd
SID334	0,02	0,59	775	14	16	Lonp1
SID343	0,00	0,63	562	10	13	Lonp1
BID274	0,04	1,50	202	4	8	Mccc1
SID1261	0,04	1,63	42	1	2	Mdh1
BID372	0,02	0,58	102	3	6	Me1
BID1266	0,00	1,69	73	1	5	Park7
SID1435	0,05	0,61	39	1	3	Pnp
SID1241	0,04	1,99	106	2	10	Rgn
BID1272	0,04	2,37	95	3	11	Rpl9
SID1047	0,02	0,66	157	3	10	Serpinb6

BID566	0,01	1,43	73	1	3	Sf3b4
BID1063	0,01	0,65	40	1	3	Slc25a5
SID1574	0,04	1,43	41	1	3	Star
SID1586	0,00	1,44	120	3	12	Star
BID1176	0,04	1,29	36	1	4	Star
SID933	0,01	1,26	173	4	10	Suclg2
BID853	0,00	1,61	175	3	10	Taldo1
BID1296	0,03	1,98	132	2	12	Tfam
SID1219	0,04	1,76	160	3	22	Txnl1

Protein name
Short-chain specific acyl-CoA dehydrogenase, mitochondrial
Acetyl-CoA acetyltransferase, cytosolic
Acyl-coenzyme A thioesterase 13
Actin, cytoplasmic 1
Actin, cytoplasmic 1
Adenosylhomocysteinase
Adenosylhomocysteinase
Serum albumin
Serum albumin
4-trimethylaminobutyraldehyde dehydrogenase
Annexin A11
Coatomer subunit delta
ATP synthase subunit gamma, mitochondrial
F-actin-capping protein subunit alpha-1
Chloride intracellular channel protein 1
Clathrin light chain A
Cystatin-B
Cholesterol side-chain cleavage enzyme, mitochondrial
Cholesterol side-chain cleavage enzyme, mitochondrial
Cytochrome P450 11B2, mitochondrial
tRNA-splicing ligase RtcB homolog
Elongation factor 1-delta
Eukaryotic initiation factor 4A-III
Fibrinogen beta chain
Glyoxalase domain-containing protein 4
Glyoxalase domain-containing protein 4
Guanine nucleotide-binding protein subunit beta-4
Heterogeneous nuclear ribonucleoprotein A3
Heterogeneous nuclear ribonucleoprotein D0
60 kDa heat shock protein, mitochondrial
60 kDa heat shock protein, mitochondrial
60 kDa heat shock protein, mitochondrial
Mitochondrial inner membrane protein
Inositol monophosphatase 1
Isovaleryl-CoA dehydrogenase, mitochondrial
Lon protease homolog, mitochondrial
Lon protease homolog, mitochondrial
Methylcrotonoyl-CoA carboxylase subunit alpha, mitochondrial
Malate dehydrogenase, cytoplasmic
NADP-dependent malic enzyme
Protein DJ-1
Purine nucleoside phosphorylase
Regucalcin
60S ribosomal protein L9
Serpin B6

Splicing factor 3B subunit 4
ADP/ATP translocase 2
Steroidogenic acute regulatory protein, mitochondrial
Steroidogenic acute regulatory protein, mitochondrial
Steroidogenic acute regulatory protein, mitochondrial
Succinyl-CoA ligase [GDP-forming] subunit beta, mitochondrial
Transaldolase
Transcription factor A, mitochondrial
Thioredoxin-like protein 1
